# More than Antibiotics: Latest Therapeutics in the Treatment and Prevention of Ocular Surface Infections

**DOI:** 10.3390/jcm11144195

**Published:** 2022-07-19

**Authors:** Ming-Cheng Chiang, Edward Chern

**Affiliations:** 1 niChe Lab for Stem Cell and Regenerative Medicine, Department of Biochemical Science and Technology, National Taiwan University, Taipei 10617, Taiwan; csmucell1223@gmail.com; 2 Research Center for Developmental Biology and Regenerative Medicine, National Taiwan University, Taipei 10617, Taiwan

**Keywords:** ocular surface infection, probiotics, stem cell therapy, ocular drug delivery systems, siRNA therapy

## Abstract

Ocular surface infections have been common issues for ophthalmologists for decades. Traditional strategies for infection include antibiotics, antiviral agents, and steroids. However, multiple drug-resistant bacteria have become more common with the prevalence of antibiotic use. Furthermore, an ideal treatment for an infectious disease should not only emphasize eliminating the microorganism but also maintaining clear and satisfying visual acuity. Immunogenetic inflammation, tissue fibrosis, and corneal scarring pose serious threats to vision, and they are not attenuated or prevented by traditional antimicrobial therapeutics. Herein, we collected information about current management techniques including stem-cell therapy, probiotics, and gene therapy as well as preventive strategies related to Toll-like receptors. Finally, we will introduce the latest research findings in ocular drug-delivery systems, which may enhance the bioavailability and efficiency of ocular therapeutics. The clinical application of improved delivery systems and novel therapeutics may support people suffering from ocular surface infections.

## 1. Introduction

The ocular surface consists of the conjunctiva, cornea, lacrimal gland, lacrimal drainage apparatus, and associated eyelid structures. It contacts the outer environment directly; thus, it is more frequently exposed to pathogens including bacteria, viruses, parasites, or fungi. Ocular surface infections include keratitis, blepharitis, conjunctivitis, dacryocystitis, and canaliculitis. These infectious disease are caused by the invasion and growth of pathogens involving any part of the ocular surface. Antimicrobials have been used for ocular infection for decades; however, ever more frequent reports of multiple drug-resistant species such as multidrug-resistant *Pseudomonas aeruginosa* (MDR-PA) and methicillin-resistant *Staphylococcus aureus* (MRSA) have been noted to be accompanied by the increased prevalence of antibiotic use [1,2]. Drug resistance in herpes simplex virus and cytomegaly virus have also been commonly noted [3,4]. Furthermore, the current antimicrobials are made in the form of eye drops, which have poor bioavailability due to fast elimination from the lacrimal drainage system and the burst of drug release on the ocular surface.

Despite the prevalence of broad-spectrum antimicrobials, vision impairment is still a serious sequela in many cases that is caused by immunogenetic inflammation. Tissue fibrosis, scarring, and corneal angiogenesis are commonly seen following ocular surface infections [5,6,7,8]. In our observation, recently there has been a shift in focus on antimicrobials to an emphasis on alternative therapies including stem-cell therapy, gene therapy, probiotics, and novel drug-delivery systems with an aim of not only combatting pathogens but exerting increased drug efficiency, reducing immunogenetic inflammation, and preserving visual acuity.

## 2. Probiotics

The ocular surface microbiota (OSM) are known as polymicrobial communities including bacteria, viruses, fungi, and archaea that inhabit the ocular surface, shaping the microenvironment and playing a role in metabolism, disease development, and immunomodulation [9]. Previous studies have indicated a relationship between the OSM and ocular surface infections. Lee et al., found a significant difference in the OSM between patients with and without blepharitis; Prashanthi et al., indicated that dysbiosis in the ocular fungal microbiome is related to fungal keratitis [10,11]. Zhou et al., recruited 210 residents in Gambia, 105 with healthy conjunctivae and 105 with clinical signs of trachoma, and found decreased diversity and an increased abundance of *Corynebacterium* and *Streptococcus* in participants with signs of trachoma [12].

Although whether alterations in the OSM are causes or outcomes of ocular surface infections remains unknown, another question is whether alterations or improvements in the OSM could serve as a treatment for infection. Kugadas et al., topically applied *Staphylococcus* spp., the predominant bacteria in the OSM, to the ocular surface of germ-free (GF) mice lacking OSM that were susceptible to *Pseudomonas aeruginosa*-induced keratitis. Two weeks later, the mono-colonized GF mice regained resistance to *P. aeruginosa*, indicating that alterations in the OSM could have dramatic impacts on host immunity [13]. In addition to the OSM, human gut microbiota (HGM), which are far from the eyes, have been found to be related to ocular surface health, known as the gut-eye axis [14,15]. An increased abundance of the pathogenic fungi *Aspergillus* and *Malassezia* were noted in fecal samples collected from patients with bacterial keratitis [16]. Mice treated with oral antibiotics were also more susceptible to ocular *P. aeruginosa* infection due to interference in the gut microbiota [13]. Novel therapies based on these findings have been considered. “Probiotics” are live nonpathogenic microorganisms administered to improve microbial balance, particularly in the gastrointestinal tract, and beneficially affect the host [17,18]. They are expected to ameliorate ocular surface health through improving the human gut microbiota [19].

The antimicrobial effects of probiotics including the secretion of bacteriocin, ribosomally synthesized peptides, or proteins with antimicrobial activity and competition for nutrition and space have been clearly indicated [20,21,22]. Five species belonging to *Lactobacillus* (*L. rhamnosus*, *L. brevis*, *L. plantarum*, *L. acidophilus*, and *L. rhamnosus*) were found to reduce the biofilm formation of *Bacillus* spp. [23]; the growth of *Neisseria gonorrhea* and herpes simplex virus -2, which contribute to neonatal conjunctivitis, were significantly inhibited by *L. rhamnosus* strain L60 and *L. crispatus*, respectively [24,25]. Human toxocariasis has ophthalmic involvement, known as ocular larva migrans, caused by *Toxocara canis*. A decreased *T. canis* burden was noted in mice inoculated with *Enterococcus faecalis* CECT 7121 before infection compared with those without inoculation [26].

However, it has never been proven that bacteriocin or other antimicrobial substances can reach the eyes; competition between commensal bacteria and pathogens may only happen in local areas, suggesting that oral administration or fecal transplantation may not currently be practical. In addition, the above-mentioned studies were mostly conducted in cells or mouse models. With regard to topical usage, probiotic eyedrops have been reported and utilized in vernal keratoconjunctivitis but not yet in ocular surface infections [27]. It is necessary to investigate the relationship and interaction between different probiotics and pathogens and then to establish the “antimicrobial spectrum” of each strain in order to select the most efficient probiotic strain case by case. Future research is also required to compare the efficiency, safety, and cost-performance ratio of probiotics and traditional antimicrobial agents. Though the application of probiotics to ocular surface infections has its limitation now, it has the undeniable merit of offering a novel treatment with great potential.

## 3. Toll-like Receptors (TLRs) and Prevention of Ocular Infection

**Figure 1:**
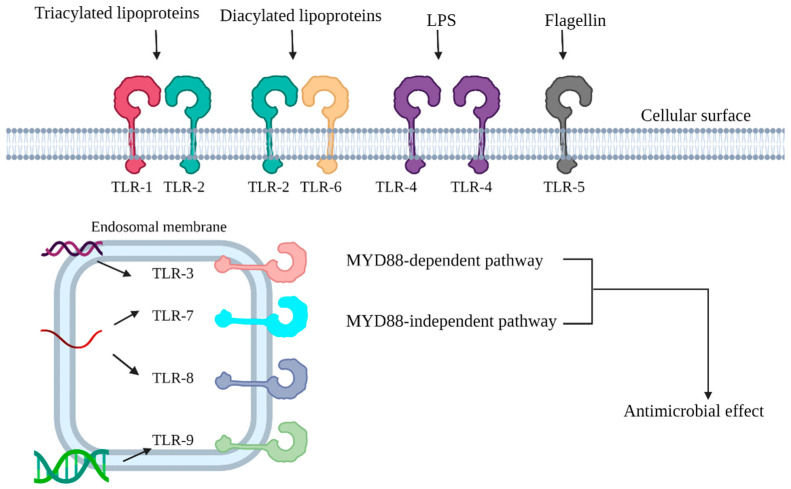
Each Toll-like receptor has its own function. The TLR1/2 complex recognizes the triacylated lipoprotein commonly found in Gram-negative bacteria, and the TLR2/6 complex recognizes the diacylated lipoprotein synthesized by Gram-positive bacteria [35,36,37]. LPS serves as a specific ligand for TLR4, and flagellin can bind to TLR5 [38]. With regard to TLRs located on the endosomal membrane, TLR3 recognizes dsDNA [39], and both TLR7 and TLR8 can recognize ssRNA [40]. TLR9 can bind to CpG-containing DNA [41]. Activated TLRs then trigger a downstream cascade and contribute to antimicrobial effects.

TLRs are transmembrane glycoprotein receptors belonging to the family of pattern cognition receptors (PRRs) that are capable of recognizing pathogen-associated molecular patterns (PAMPs), which are derived from microorganisms and include lipopolysaccharides (LPSs), lipoteichoic acid in Gram-positive bacteria (GPB), lipoproteins, and mycobacterial lipoglycans [28,29,30,31]. TLRs can be divided into two groups depending on their cellular localization and PAMP ligands. TLR1, TLR2, TLR4, TLR5 and TLR6 are categorized together as they are located on cellular surfaces and recognize microbial membrane components. The other group is composed of TLR3, TLR7, TLR8, and TLR9, which are located in intracellular vesicles and recognize microbial nucleal acids [32]. Each TLR has its own specific characteristics and functions, sensitizing and binding to different PAMPs. Once bound to a specific ligand and activated, TLRs can trigger downstream signaling pathways including myeloid differentiation primary response 88 (MYD88)-dependent and independent pathways. The MYD88-dependent pathway leads to the quick activation of mitogen-activated protein kinase (MAPK) and NF-κB, and ultimately to induction of proinflammatory cytokines; the other pathway is related to IFN-β secretion and dendritic-cell maturation, both of which contribute to innate immunity (Figure 1) [33,34].

However, TLRs remain stable most of the time. TLR2 and TLR4 are expressed intracellularly in human cornea epithelial cells, and TLR5 is located in wing cells and the basal cell layer instead of the outermost part of the cornea [38,42]. The expression of myeloid differentiation factor 2 (MD-2), an essential component for LPS-TLR4 signaling, is deficient in corneal epithelial cells [43]. By these mechanisms, TLRs remain silent to non-pathogenic bacteria on the ocular surface but generate TLR-induced immunity once the integrity of corneal surface is broken.

Current studies have attempted to apply TLRs to ocular surface infections. The topical application of flagellin, a ligand for TLR5, before *P. aeruginosa* inoculation has been shown to attenuate the clinical symptoms of *P. aeruginosa* keratitis, decrease bacterial burden and induce antimicrobial peptide secretion in B6 mouse corneas. As for clinical applications, the addition of low-dose purified flagellin in a contact-lens solution can serve as a prophylactic strategy for *P. aeruginosa* keratitis as it is commonly noted in contact-lens wearers [44]. Bacterial endophthalmitis is a serious complication of ocular surgery that may lead to significant vision loss or even blindness. Kumar et al., administered Pam3Cys, a synthetic ligand of TLR2, or phosphate-buffered saline intravitreally to C57BL/6 mice 24 h prior to *Staphylococcus aureus* inoculation and compared the severity of endophthalmitis by electroretinography, histological examinations, and bacterial load. The outcome revealed a significantly lower bacterial load, preserved retinal structural integrity and normal response toward light stimulation on electroretinography in the Pam3Cys-treated group [45]. Endotoxin tolerance refers to a phenomenon in which cells or organisms exposed to low concentrations of endotoxin develop a transient unresponsive state and are unable to respond to further challenges with endotoxin, aiming to prevent extensive tissue damage or pathological states such as sepsis [46]. TLRs are believed to be involved in endotoxin tolerance, and a novel therapy based on this concept has been reported [47,48]. The repeated low-dose administration of a TLR4 agonist LPS successfully decreases hosts’ response to subsequent LPS stimulation, again indicating the prophylactic properties of TLRs in ocular infections [46].

During an ocular surface infection, the immune response is a double-edged sword; it recruits immune cells and produces proinflammatory factors to eliminate pathogens, but immunogenetic inflammation around the infected site may lead to damage including tissue fibrosis, scaring, or opacity [49,50]. Khatri et al., added *P. aeruginosa* endotoxin to mice corneal epithelial cells and found a significant increase in stromal thickness and haze compared with untreated control corneas. Furthermore, stromal thickness and haze were not increased in mice with a single point mutation in the *TLR4* gene after the addition of endotoxin, suggesting that TLR4 is essential for recruitment of neutrophils and for the development of endotoxin-induced stromal disease [51]. TLR-related proinflammatory factors increase vascular permeability and exert strong chemotactic drift, leading to a decreased blood–retinal barrier and the gathering of neutrophils to the infection site, ultimately amplifying the inflammation. The expression levels of TLR-related proinflammatory factors were found to be positively correlated with the severity of infectious endophthalmitis [52].

Although current studies have consistently indicated the importance of TLRs in ocular surface infections, it remains unknown whether the early application of specific ligands for TLRs may reduce the severity of infection or enhance immunogenic inflammation and the related tissue damage. In addition, intravitreal injection carries potential risks of trauma, infection, or retinal detachment, which should be taken into consideration [53]. Regarding topical use or other products (eyedrops, contact-lens solution), the difficulty lies in how to preserve the ligands in these products and which kind of ligand should be utilized. The potential of its use clearly needs further exploration.

## 4. Stem-Cell Therapy

Stem cells have the capacity to both self-renew and give rise to differentiated cells [54]. In the past, stem cells were only found in the umbilical cord, and their development was limited due to ethical concerns and rarity. However, stem cells are now more readily available since induced pluripotent stem cells (iPSCs) and mesenchymal stem cells (MSCs) are both isolated from patients’ mature tissue; the former are derived from well-differentiated somatic cells, and the latter are multipotent stromal cells isolated from bone marrow, adipose tissue, dental pulp, and amnionic fluid [55,56].

### 4.1. MSCs—Tissue Repair

As mentioned above, immunogenic inflammation can cause tissue injury that may reduce visual acuity even if the infection is cured. MSCs can migrate, integrate, and differentiate into tissue-specific cells to repair damaged tissues which release injury signals [57,58]. Castanheira et al., intravitreally transplanted MSCs into laser-damaged rat eyes. The grafted cells survived for eight weeks with the expression of rhodopsin and parvalbumin, a cell marker of bipolar and amacrine cells, indicating the migration, integration and differentiation ability of MSCs [59]. However, the importance of MSCs in tissue repair lies not only in migration and differentiation. The paracrine effect refers to the interaction and signaling passage between one cell and another. MSCs can release secretomes, consisting of microvesicles, exosomes, proteins, and cytokines, to partially repair injured tissue or modify cell behavior [60,61,62]. Several infectious diseases, such as viral, bacterial, and fungal keratitis, trachoma, and *Acanthamoeba* infection, may cause fibrosis on the ocular surface, leading to reduced visual acuity, opacity on the visual axis, or astigmatism [5,6,7]. Fibrogenesis is related to secretion of types I and III collagen and TGF-β1, which are suppression targets of MSCs [63,64]. MSCs have been proven to be able to secrete hepatocyte growth factor (HGF), which is capable of reducing both types I and III collagen secretion [65,66,67]. Nagaya et al., compared mouse myocardium with and without MSC treatment, and the outcome revealed attenuated MMP-2 and MMP-9, which are matrix metalloproteinases that participate in fibrosis development in tissue remodeling, in the treated group [68,69]. Zhou et al., utilized a mouse model of fungal keratitis to compare the treatment outcomes of mice that were administered an antifungal agent alone and those that were treated with both an antifungal agent and a subconjunctival injection of umbilical MSCs. The latter group showed a reduced corneal scar-formation area and corneal opacity along with downregulated fibrosis-related factors [70]. Furthermore, MSCs have also been shown to inhibit TGF-β1 signaling, again suggesting their antifibrosis characteristics and capability to reduce scarring [71].

### 4.2. MSCs—Antimicrobial Properties

MSCs secrete antimicrobial substances when challenged with pathogens. Krasnodembskaya et al., indicated that MSC-treated conditioned medium exhibited a marked inhibition of bacterial growth when compared with medium without treatment. One of the factors responsible for the antimicrobial activity of MSCs is the human cathelicidin antimicrobial peptide, hCAP-18/LL-37, which has been found to be increased in MSCs after bacterial challenge. In a mouse model of *E. coli* pneumonia, intratracheal administration of MSCs was found to inhibit bacterial growth, while mice treated with MSCs and a neutralizing antibody to LL-37 showed weakened bacteria reduction [72]. Another study utilizing microarray analysis identified elevated expression of β-defensin 2 (BD2) in MSCs after *E. coli* exposure. TLR-4-knockout MSCs were also found to restore antimicrobial activity with BD2 supplementation, illustrating that BD2 secreted by the MSCs via the TLR- 4 signaling pathway was a crucial factor for the microbicidal effects of MSCs [73]. Lipocalin and hepcidin were also recognized as antimicrobial substances secreted by MSCs. Lipocalin can inhibit bacterial growth by interrupting iron transportation [74,75], and hepcidin restricts iron availability to display bacteriostatic activity [76,77]. Besides, Meisel et al., indicated a broad-spectrum antimicrobial capacity of MSCs against clinically relevant bacteria, protozoal parasites and viruses in response to inflammatory cytokines [78].

### 4.3. MSCs—Immunomodulation

In the current antimicrobial strategy for ocular surface infections, short-term treatment with immunomodulators such as corticosteroids or mitomycin C combined with broad-spectrum antimicrobial agents may cure infection and avoid scarring or fibrosis secondary to immunogenic inflammation [79,80]. However, MSCs exhibit anti-inflammatory and immunomodulatory properties in ocular disease.

When situated in an inflammatory microenvironment, MSCs can suppress the maturation and differentiation of CD4+ and CD8+ T cells, B cells, and natural killer cells by releasing mediators including IDO, IL-6, TNF-α–stimulated gene 6 protein (TSG-6), nitric oxide (NO,) and PGE2 [81,82,83,84,85,86]. Once engulfed by host macrophages, MSCs release IDO, a heme-containing enzyme to facilitate the differentiation of monocytes into interleukin (IL)-10-secreting M2 immunosuppressive macrophages to inhibit T-cell proliferation [87,88]. MSCs also facilitate the proliferation of tolerogenic dendritic cells, which may lead to T-cell unresponsiveness and the generation of regulatory T (Treg) cells [89,90]. Monocyte chemoattractant protein-1 (MCP-1) is a chemokine able to induce migration and infiltration of monocytes/macrophages to inflammatory sites [91,92]. Yu et al., found that MSC-derived exosomes decrease MCP-1 mRNA expression in cultured retinal cells and that mice with laser retinal injury expressed a lower MCP-1 after the intravitreal injection of MSCs [93]. Hermankova et al., intravitreally administered the proinflammatory cytokines IL-1β, TNF-α, and IFN-γ to mice, which then induced the upregulation of IL-1α, IL-6, and VEGF in their retinas. However, by the intravitreal injection of MSCs, all of the above-mentioned proinflammatory factors were significantly decreased [94].

### 4.4. MSCs—Limitations and Future Directions

**Figure 2:**
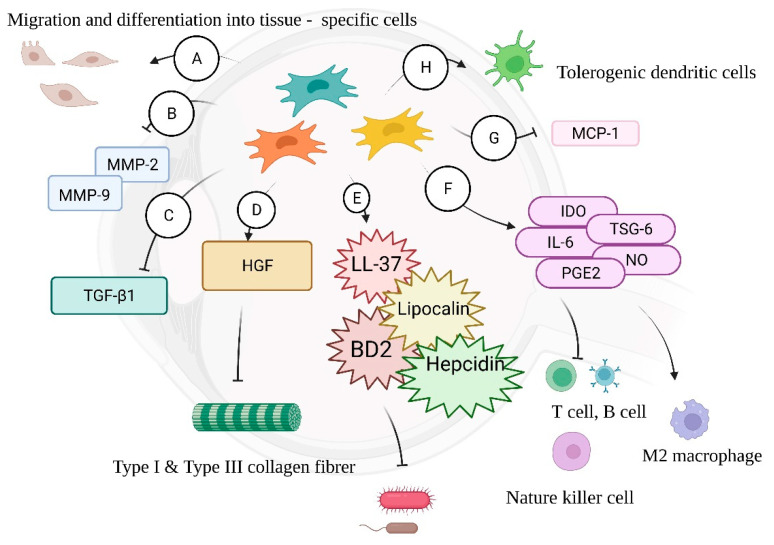
(A) MSCs can migrate to a site of inflammation and differentiate into tissue-specific cells for repair. (B) MSCs inhibit the expression of MMP-2 and MMP-9, attenuating scar formation. (C) Inhibition of TGF-β1 signaling reduces fibrogenesis. (D) MSC-derived HGF reduces type I and type III collagen fibers, which contribute to scarring. (E) Antimicrobial molecules including LL-37, lipocalin, BD2, and hepcidin secreted by MSCs can inhibit bacterial growth. (F) IDO, TSG6, IL-6, PGE2, and NO can exert immunomodulation, inhibiting proliferation of T cell, B cells, and natural killer cells and inducing M2 macrophage differentiation. (G) MCP-1, which induces immune-cell infiltration, is inhibited by MSCs. (H) Tolerogenic dendritic cells are enhanced by MSCs.

Despite having multiple functions in ocular surface infections, including scarring reduction, tissue repair, antimicrobial properties, and immunomodulation (Figure 2), the clinical application of MSCs is still facing challenges. First, once MSCs are detached from the culture plate, anoikis may happen with the loss of matrix support [95,96]. After transplantation into the eyes, an infected eye is an inflammatory microenvironment for graft MSCs in which oxygen and nutrition are depleted with the accumulation of reactive oxygen species and leukocytes [97,98]. Indeed, a portion of grafted cells may survive, but even MSCs derived from the same organ consist of a heterogenous population, increasing the uncertainty of their differentiation potential and cellular behavior [99]. Furthermore, MSC therapy requires advanced facilities, well-trained physicians, and considerable funds, which may hinder its use.

However, several strategies to improve graft-cell survival have been reported [100,101,102], and more studies are in progress. Stem-cell therapy is sure to be of considerable value to those suffering from ocular surface infections; however, more extensive research is necessary to make any definitive claims along these lines.

## 5. siRNA

Herpes simplex keratitis (HSK) is induced by herpes simplex virus (HSV) and can be divided into herpes simplex epithelial keratitis, herpes simplex stromal keratitis, and herpes endothelial keratitis depending on the depth of the viral invasion. The current management is generally centered around corticosteroid and antiviral agents. According to a guideline published by the American academy of ophthalmology, an oral or a topical antiviral agent alone is enough for herpes simplex epithelial keratitis treatment, and a topical steroid in conjunction with an oral antiviral agent is the preferred treatment strategy for herpes simplex stromal and endothelial keratitis. However, a topical corticosteroid should be avoided in the initial treatment of herpes simplex epithelial keratitis [103].

Apart from the tissue damage caused by viral infection and its related inflammation, corneal angiogenesis is an important issue in HSK, and bioactive ‘‘CpG’’ motifs of HSV DNA may be the culprit. CpG motifs are composed of unmethylated CpG dinucleotides and are prevalent in viruses and bacteria instead of humans [104]. They are taken as PAMPs that can be recognized by Toll-like receptors and activate human innate and acquired immunity [105,106]. Zheng et al., reported that oligodeoxynucleotides containing CpG motifs upregulate VEGF expression and lead to corneal angiogenesis, while neutralizing oligodeoxynucleotides suppress corneal angiogenesis by 60% [107].

**Figure 3:**
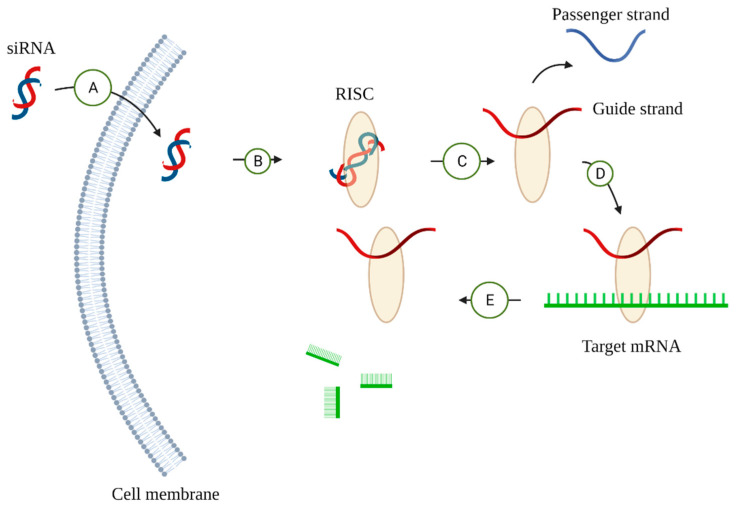
The regulatory mechanism of siRNA on gene expression. (A) siRNAs pass the cell membrane. (B) siRNAs then load into the RNA-induced silencing complex (RISC), which is part of a family of ribonucleoprotein complexes and is able to locate target mRNAs [111]. (C) RISC selects a strand with a less thermodynamically stable 5’ end and degrades the other strand, known as a passenger strand [112,113,114]. (D) The RISC–siRNA complex binds to the target mRNA. (E) The target mRNA is degraded.

Small interfering RNAs (siRNAs) are 21- to 25-nucleotide small RNAs capable of recognizing a homologous mRNA sequence and inducing its degradation, participating in post-transcriptional silencing (Figure 3) [108,109]. Kim et al., designed an siRNA targeting VEGF (mVEGFA) and two siRNAs targeting VEGF receptors (mVEGFR1, mVEGFR2) and administered them into mice systemically or subconjunctivally. The outcome revealed a significantly reduced area of angiogenesis compared with the control group, which was administered siLacZ. Although each siRNA expressed comparable angiogenic inhibition when administered separately, the mixture of three siRNAs suppressed angiogenesis more significantly. This study strongly suggested that siRNA mediated an anti-angiogenic effect with a clinically feasible delivery system to pave the way for a new wave of treatment for HSK [110].

Although this research outcome is encouraging, some difficulties remain to be resolved. siRNAs can only enter a cell via endocytosis or pinocytosis since they are polyanions and unable to directly pass through the hydrophobic cell membrane. Even approaching the cytoplasm, they may be cleaved by cytoplasmic ribonucleases or be excluded from cells via exocytosis, decreasing their intracellular concentration [115,116]. In the previously mentioned studies by Kim et al., a dose-dependent response was noted as the inhibitory area of angiogenesis was positively correlated with the siRNA dose [110]. However, the higher the siRNA concentration is, the more frequently the toxin effect may occur; this is known as the nonspecific immunostimulatory effect triggered by siRNAs and/or their delivery vehicles, leading to the stimulation of the immune system and the induction of an inflammatory response. siRNA mediated off-target silencing, which regulated unintended transcripts and led to a toxic phenotype, thereby increasing the uncertainty of its clinical use. Additionally, the off-target effect may not be eliminated by reducing the siRNA concentration [117,118]. Current studies have focused on the modification of siRNAs to minimize their immunostimulant properties, and lipid particles, lipidoids, and cyclodextrin polymers have been utilized as delivery vehicles without inducing significant toxicity [115,119,120,121]. We look forward to more applications of gene therapy to ocular surface infections.

## 6. Cysteine Protease Inhibitors

Tears are known to participate in the innate immunity of the ocular surface, which contains antimicrobials and anti-inflammatories [122]. Surfactant protein D (SP-D) plays an important role in this defensive line. SP-D can aggregate pathogens and facilitate pathogenic clearance. Compared with wild-type mice, mice with the depletion of SP-D with mannan-conjugated sepharose or anti-SP-D antibody had increased susceptibility to bacterial keratitis [123,124]. Cysteine protease is an extracellular protease secreted by bacteria that degrades SP-D [125]. Zhang et al., indicated that a cysteine protease inhibitor may ameliorate infection in wild-type mice but lead to no significant improvement in SP-D-knockout mice, suggesting that a cysteine protease inhibitor may be a potential therapeutic [126].

## 7. Drug Delivery—In Situ Gels

To combat ocular surface infections more efficiently, amelioration in antimicrobial agents is an important issue, but how they are delivered also matters. Topical instillation is the most common method of ocular drug delivery as it is noninvasive and easy. However, its bioavailability is less than 5%, and it is generally removed from the ocular surface in five minutes by the lacrimal drainage system [127,128]. An improvement in ocular drug delivery is needed to increase the efficacy of the current medications. Previous studies have demonstrated several vehicles, including ointments, inserts, and aqueous gels, to prolong drug retention on the ocular surface [129,130]. However, the above-mentioned delivery methods may either cause temporary blurred vision due to opacity on the ocular surface or lead to poor patient compliance due to inconvenience or discomfort.

In situ gels are preserved as solutions or suspensions and undergo rapid sol-to-gel transformation upon contacting the ocular surface, triggered by external stimuli such as temperature, pH, and ionic strength [131,132,133]. As viscoelastic gels form on the ocular surface in response to environmental stimulation, they slowly release drugs under physiological conditions to enhance bioavailability, minimize systemic absorption, and extend the residence time of drugs [134,135]. Chitosan, a natural polymer obtained by the deacetylation of chitin, is the most common polymer used in in situ gels, and it consists of repeating units of N-acetyl-d-glucosamine and d-glucosamine linked by β-(1–4) glycosidic bonds, making it is less toxic, biocompatible, and biodegradable. Its properties are strongly affected by its molecular weights, ranging between 3800 and 20,000 Da, and its degree of deacetylation [136,137]. The current applications are based on chitosan combined with various artificial or natural materials to create in situ gels with different properties.

### 7.1. pH-Sensitive In Situ Gels

The pH value changes the chemical and physical properties of pH-sensitive in situ gels as acidic groups and basic groups are deprotonated and pronated in response to pH changes in the surrounding environment [138]. Carbopol is a polyacrylic acid derivative serving as a viscosity-enhancing agent when combined with chitosan. Gupta et al., indicated that a 0.4% *w/v* carbopol/0.5% *w/v* chitosan-based in situ gelling system (ISGF3) is an ideal vehicle for Timolol maleate (TM) delivery, which is an antiglaucomatous drug, since it remained as a liquid in room-temperature air at a pH value of 6.0 and underwent rapid transition into the viscous gel phase at the pH of the tear fluid (pH 7.4). TM prepared with 0.4% carbopol without chitosan released almost 90% within 8 h, while formulations with undialyzed ISGF3 gels released only 60.9% of TM after 24 h, indicating the carbopol–chitosan mixed regimen prevented premature drug release much better than carbopol alone [139]. Gatifloxacin is an antibiotic of the fluoroquinolone family that inhibits the topoisomerase of bacteria [140]. A pH-sensitive in situ gel formula consisting of hydroxypropyl methylcellulose and carbopol has been developed for gatifloxacin delivery, and its gelation occurred at pH 6.9–7.0 with sustained drug release for more than eight hours. Additionally, it was a stable product as no definite changes were observed in its intactness for 12 days after the study [141]. Makwana et al., also evaluated a pH-sensitive in situ gel carrying ciprofloxacin that showed significantly improved ocular bioavailability [132].

### 7.2. Thermosensitive In Situ Gels

Temperature-sensitive in situ gels are preserved as a liquid in room temperature and transition into gels with temperature changes. A chitosan/β-glycerophosphate (CS–GP) mixture is the most commonly used polymer [142]. When carrying therapeutics with low water solubility, such as latanoprost and ferulic acid, hydrogels may release them more slowly than water-soluble therapeutics such as timolol maleate [143,144,145]. β-glycerophosphate serves as a secondary crosslinker to enhance the function of chitosan by reducing the pore size within the hydrogel networks [146]. Chitosan hydrogels without any addition failed to prolong drug release, releasing 100% of the drug in one hour [147,148]. In addition, improvement in preformulating therapeutics is a new strategy to prolong drug release. Fabiano et al., introduced chitosan-based nanoparticles medicated with 1.25 mg/mL 5-FU into a thermosensitive ophthalmic hydrogel and demonstrated that the hydrogel with nanoparticles showed significantly slower drug release than 5-FU hydrogel not containing nanoparticles [149]. A similar concept has been applied to antimicrobials. Oxytetracycline-loaded gelatin–polyacrylic acid nanoparticles in poloxamer-407 solution, which is a triblock copolymer with thermoreversible properties, released 93% of oxytetracycline after 24 h, while a pure drug from the dialysis membrane in simulated tear fluid released almost 100% in the first two hours. In addition, it exerted satisfying antimicrobial activity toward *P. aeruginosa* keratitis [150]. Kong et al., obtained levofloxacin chitosan microspheres using a microfiltration membrane with a pore size of 0.02 µm. The outcome revealed that the levofloxacin chitosan microspheres could prevent a burst release during the initial phase and extend the contact duration of levofloxacin with the eyes [148]. In summary, improvement and modification in both vehicles and therapeutics can enhance bioavailability and thus reduce administration frequency, thereby enhancing patient compliance.

### 7.3. Ion-Sensitive In Situ Gels

An ion-sensitive gelling system is triggered by alterations in ion concentration, and is able to crosslink with the cations in the tear fluid to form a gel on the ocular surface, improving drug retention and bioavailability [151]. Gellan gum (Gelrite^®^), a member of the bacterial polysaccharides, is a commonly used material, known to undergo a phase transition in presence of mono-, di- and trivalent cations including potassium, magnesium, calcium and aluminum [152,153,154]. It is a linear polymer with a repeating sequence consisting of one α-l-rhamnose, two β-d-glucose, and one β-d-glucuronate [155,156].

However, a drug-delivery system is never made from gellan gel only; it is prepared with different compounds in various proportions. Several attempts have been made to figure out the ideal formulation for each ocular therapeutic. Geethalakshmi et al., developed an ion-sensitive in situ gel (0.2 g of brimonidine tartrate, 0.6 gm gram of gellan gum and benzalkonium chloride 0.02% *w/v* in 100 mL distilled deionized water) that had a better gelling capacity, less ocular surface irritation and a more satisfying drug-release profile as it could sustain the brimonidine action for 8 h, compared with other formulations containing different proportion of gellan gum [157]. Sun et al., compared three formulations with different proportions of gellan gum for ocular brinzolamide delivery, and the outcome revealed that the formulation compromised of 1% brinzolamide, 1% gellan gel, 5% mannitol and 0.01% chlorhexidine acetate extended drug retention on the ocular surface the most significantly [158].

With regard to ocular surface infections, fungal keratitis has been a difficult issue for physicians for a long time. Antifungal therapeutics are now available in ion-sensitive gelling systems. Fernández-Ferreiro et al., mixed hydroxypropyl-β-cyclodextrin (HBC) with fluconazole as an inclusion complex and incorporated it into gellan-gum-based in situ gels. It had significantly better bioadhesive properties, demonstrated a more prolonged drug release for approximately 4 h, and permitted the release of a higher quantity of the antifungal than gels without HBC [159].

Terbinafine-loaded nano-emulsion gels that contained particles at the nano level have been described. A specific regimen of F31 (Miglyol^®^ 812, Cremophor^®^ EL: polyethylene glycol 400 (1:2) and water (5, 55 and 40%, *w/w*, respectively)) was found to release terbinafine at a controlled rate, starting with 10% at the first hour and continuing with 10% increments every hour. F31 also reached a higher maximum drug dose on the ocular surface and exerted better bioavailability than the oily drug solution without ocular irritation [160].

Besides gellan gum, other materials have been applied to ion-sensitive in situ gels. Sodium alginate is the sodium salt of alginic acid, which is commonly found in capsular of soil bacteria, composed of α-l-guluronic (G) and β-d-mannuronic (M) residues. The gelation of alginate is led by interactions between calcium ions and G residues [161]. Sodium alginate has been broadly applied to ocular drug delivery as it possesses mucoadhesive properties without causing blurred vision, and it extends drug retention time on the ocular surface [162]. Liu et al., combined sodium alginate and hydroxypropyl methyl cellulose (HPMC), a viscosity-enhancing agent, in different proportions, attempting to enhance the retention time of gatifloxacin on the ocular surface. The outcome revealed that a formulation (A8) with 1% sodium alginate and 2% HPMC was found to achieve a 2.6-fold improvement in precorneal retention time, with great gelling capacity and no ocular irritation, compared with the conventional gatifloxacin ophthalmic solution. Additionally, formulation A8 had the smallest burst release. It released 17.2% of gatifloxacin after 30 min, while the gatifloxacin ophthalmic solution released almost all of it in 30 min [163]. Furthermore, the ocular delivery of moxifloxacin, daptomycin and linezolid have been beneficial to the application of the alginate-based delivery system [164,165,166]. Pectin is a natural polymer constituting the cell walls of most plants, which undergoes gelation upon binding to calcium cations [167]. Pectin has been used in ocular drug delivery combined with other viscosity enhancers. It had been utilized as a drug carrier of azithromycin, exhibiting prolonged drug release, a stable gelation phase, and effective inhibition of bacterial growth [168].

Despite several advantages including sustained drug release, increased bioavailability, and decreased visual opacity, the clinical application of in situ gels still faces some obstacles that do not have immediate solutions. The increased viscosity of an in situ gel enhances the drug adhesion to the ocular surface but may cause blurred vision for patients, and further detailed research is required to find an appropriate viscosity with a satisfying drug-release profile and a minimal influence on vision. Ocular irritability and toxic effects caused by repeated dosing and long-term usage should be thoroughly investigated. Future work will hopefully clarify these issues to maximize the effectiveness of these systems.

## 8. Drug Delivery—Ocular Inserts

Ocular inserts are defined as sterile, thin, multilayered, drug-impregnated, either insoluble, soluble, or bio-erodible devices placed into the cul-de-sac or conjunctival sac to enhance bioavailability and sustain the release of medication on the ocular surface. Drug release from ocular inserts can mainly be categorized in three ways: diffusion, bioerosion and osmosis. Diffusion ocular inserts are composed of a central drug reservoir enclosed in semi-permeable or micro-porous membranes, and the drugs are continuously released at a controlled rate onto the ocular surface through the pores or the membrane. Bioerosion inserts are comprised of a matrix of bioerodible material in which the drug is dispersed. Bioerosion happens when the insert is infiltrated by tears, leading to the controlled sustained release of the drug [169]. Osmosis ocular inserts are generally made up of a central part, either divided into two separated compartments or composed of a single one, with a peripheral part surrounding it. In the first case, the central part is divided into a drug compartment surrounded by an elastic impermeable membrane and an osmotic compartment containing osmotic solutes, which is surrounded by a semi-permeable membrane. In the second case, the central part is simply the drug with or without an osmotic solute dispersed through a polymeric matrix; namely, the drug is surrounded by the polymer as discrete small deposits and the peripheral parts of the above-mentioned osmosis ocular inserts cover films that consist of insoluble semipermeable polymers. The mechanisms of drug release in single- or double-compartment osmosis ocular inserts are different. In double-compartment osmosis inserts, once contacting the ocular surface, tears diffuse into the osmotic compartment, leading to an osmotic pressure that stretches the elastic membrane and contracts the drug compartment so that the drug is pushed out through the single drug-release aperture. In the single-compartment device, the tear fluid diffuses into the peripheral part through the semipermeable polymeric membrane, wets the drug and induces its dissolution. The solubilized deposits then create hydrostatic pressure against the polymer matrix, causing its rupture and creating apertures for drug release [170,171]. Various types of ocular inserts are now available, including membrane-bound ocular inserts, filter paper strips, collagen shields, etc. [172]. For ocular surface infection, ciprofloxacin, fluconazole, azithromycin, acyclovir and other antimicrobial agents have been successfully carried by ocular inserts [173,174,175,176].

However, ocular inserts have their limitations. Regardless of the type of ocular insert, it may cause foreign body sensations for patients and induce lacrimation, lowering the drug concentration [177] and reducing patient compliance, especially in children and incompetent persons.

## 9. Drug Delivery—Nanofiber

Nanofibers are drug carriers with diameters in the nanometer range. One gram of nanofibers with 100 nm diameter could offer a surface area of 1000 square meters. The large surface area of nanofibers can enhance mucoadhesion and improve the bioavailability of drugs. Besides, nanofibers can provide a large drug-loading capacity, which is a serious limitation in other drug-delivery system. Nanofibers can convert drugs from a crystalline state to an amorphous state, enhancing their solubility, especially for drugs with a narrow absorption window [178].

Nanofibers can be processed into different products in order to exert its advantages in drug delivery. Nanofiber-based ocular inserts have been used as effective drug carriers. Taghe et al., prepared azithromycin-loaded chitosan/polyvinylalcohol/polyvinyl pyrrolidone (CS/PVA-PVP) nanofibers by electrospinning. Due to the large drug-loading capacity of nanofibers, the ocular inserts became smaller in size, which may lower discomfort for patients. A group of nanofibers composed of 3% PVA and 1% PVP with Glutaraldehyde, a strong crosslinker, showed 57.59 ± 1.78% of drug release after 142 h, while a traditional azithromycin solution released all of the drug within 20 h. The nanofiber-based ocular inserts also exerted satisfying antimicrobial efficacy with good biocompatibility, and no cytotoxicity or ocular irritation was noted in any of the samples [175]. Similar findings have been published and applied to other antibiotics such as moxifloxacin, gentamycin and ofloxacin [179,180,181].

Some suture lines are known to be antibiotic-eluting, but antibacterial coatings are only available with absorbable threads (sized 6–0), which do not meet the requirements of ocular surgeries. The problems lie in meeting the diameter requirements, loading a sufficient amount of the drug, and maintaining the strength of the suture lines. However, the nanofiber may lead a revolution. Parikh et al., utilized polycaprolactone (PCL) to conduct a 10–0, levofloxacin-eluting, nanofiber-based multifilament suture line. The individual nanofibers had an average width of 729.9 ± 246 nm. Among all the nanofiber-based sutures coated with different concentrations of levofloxacin, only the suture coated with 8% levofloxacin surpassed the minimum breaking-strength specification of the United States Pharmacopeia (U.S.P). Three parallel scratches were made across the surface of the rats’ corneas, then the corneas were implanted with different sutures and *Staphylococcus aureus*. Compared with Vicryl^®^, Nylon alone, and Nylon with a daily or single levofloxacin eye drop, the cornea that was implanted with PCL/8% levofloxacin had the significantly lowest corneal bacterial load among all of the samples (*p* < 0.05). It is an interdisciplinary outcome, indicating a novel strategy for the prevention of ocular surface infection after ocular surgery or trauma, again demonstrating the unlimited and promising potential of nanotechnology in ophthalmology.

## 10. Drug Delivery—Contact Lenses

In addition to in situ gels, contact lenses can be used to extend drug release on the ocular surface. An ideal drug-releasing contact lens should meet several criteria including adequate light transmission, comfortable surface wettability, and bactericidal antimicrobial concentration. Silicone hydrogel contact lenses have been made for ciprofloxacin release, and these have similar material properties to commercial contacts. Although a shorter wave transmission was noted, over 80% light transmission between 400 nm and 700 nm was permitted. In a rabbit model with bacterial keratitis, the number of colony-forming units (CFUs) on the corneal surface with modified contact lenses showed no significant difference compared with those treated with eye drops, suggesting that drug-eluting contact lenses may serve as supplementary treatments in ocular surface infections [182]. Moxifloxacin has been loaded onto chitosan-based contact lenses. The in vivo drug-release profile revealed that drug-eluted contact lenses extended the drug-release time for more than 10 h compared with eye drops, and the moxifloxacin concentration on the ocular surface (cornea, sclera, and lens) was significantly increased. In addition, drug-eluted contact lenses had better antimicrobial activity for *S. aureus*. Much fewer CFUs were noted on the corneal epithelium than those treated with traditional eye drops [183].

With their satisfying drug-release pattern and distribution as well as efficient antimicrobial activity, drug-eluted contact lenses are an exciting and revolutionary step in the treatment of ocular surface infections. These contact lenses can also be utilized without concern for the side effects of stem-cell therapy or other novel medications since they are eluted with antimicrobials that have been commonly used for decades. Drug-eluted contact lenses may not be suitable for children or patients with poor compliance, and a contact lens may be an irritable foreign body for an infected eye. However, these limitations do not invalidate its potential for future applications.

## 11. Conclusions

The thorough treatment of ocular surface infections is not limited to eliminating the microorganism; it also involves the prevention of ocular scarring or opacity, minimization of infection-related tissue damage, and preservation of visual acuity to ensure patient quality of life. Fruitful outcomes in therapeutic regimens, manufacturing processes, and drug-delivery systems have been achieved, and ophthalmologists can deal with ocular surface infections via the flexible application of several novel strategies and save more eyes in the near future.
